# Hydrogen sulfide promotes immunomodulation of gingiva-derived mesenchymal stem cells via the Fas/FasL coupling pathway

**DOI:** 10.1186/s13287-018-0804-6

**Published:** 2018-03-09

**Authors:** Ruili Yang, Tingting Yu, Dawei Liu, Songtao Shi, Yanheng Zhou

**Affiliations:** 1 Department of Orthodontics, Peking University School and Hospital of Stomatology, National Engineering Laboratory for Digital and Material Technology of Stomatology, Beijing Key Laboratory of Digital Stomatology, Beijing, 100081 China; 2 Department of Anatomy and Cell Biology, University of Pennsylvania, School of Dental Medicine, Philadelphia, PA 19104 USA

**Keywords:** Cell signaling, Fas pathway, Immunity, Stem cells, Infectious diseases

## Abstract

**Background:**

Mesenchymal stem cells derived from gingiva (GMSCs) display profound immunomodulation properties in addition to self-renewal and multilineage differentiation capacities. Hydrogen sulfide (H_2_S) is not only an environmental pollutant, but also is an important biological gas transmitter in health and disease.

**Methods:**

We used an in-vitro coculture system and a mouse colitis model to compare the immunomodulatory effects between control and H_2_S-deficient GMSCs. The flow cytometry analysis was used for T-cell apoptosis and T-helper 17 (Th17) and regulatory T (Treg) cell differentiation.

**Results:**

We revealed that GMSCs exerted their immunomodulatory effect by inducing T-cell apoptosis, promoting Treg cell polarization, and inhibiting Th17 cell polarization in vitro*.* The levels of H_2_S regulated the immunomodulatory effect of GMSCs. Mechanically, H_2_S deficiency downregulated the expression of Fas in GMSCs, resulting in reduced secretion of monocyte chemotactic protein 1 (MCP-1), which in turn led to decreased T-cell migration to GMSCs mediated by MCP-1. Moreover, H_2_S deficiency downregulated the expression of Fas ligand (FasL) in GMSCs. The Fas/FasL coupling-induced T-cell apoptosis by GMSCs was attenuated in H_2_S-deficient GMSCs. Consistent with this, H_2_S-deficient GMSCs showed attenuated therapeutic effects on colitis in vivo, which could be restored by treatment with the H_2_S donor, NaHS.

**Conclusions:**

These findings showed that H_2_S was required to maintain immunomodulation of GMSCs, which was mediated by Fas/FasL coupling-induced T-cell apoptosis.

**Electronic supplementary material:**

The online version of this article (10.1186/s13287-018-0804-6) contains supplementary material, which is available to authorized users.

## Background

Gingiva-derived mesenchymal stem cells (GMSCs), isolated from easily accessible tissues of oral mucosa, possess self-renewal, multilineage differentiation, and immunomodulatory properties [1]. GMSCs exert their immunomodulatory effects via interaction with innate and adaptive immune cells. Compared to bone marrow mesenchymal stem cells (BMMSCs), GMSCs proliferated rapidly and were more stable morphologically and functionally. Systemic administration of GMSCs has been proven to promote skin wound healing [2] and mitigate chemotherapy-induced oral mucositis [3], as well as attenuating experimental colitis in the mouse model [1]. Mechanically, GMSCs display suppressive effects on proliferation and activation of peripheral blood mononuclear cells (PBMCs) [4, 5]. GMSCs induced T-cell apoptosis via a Fas/FasL pathway upon cell–cell contact [6]. GMSCs also promoted Treg cell polarization and inhibited Th17 cell polarization [1, 5, 7]. GMSC infusion has been shown to promote the survival of skin allografts by increasing the infiltration of Treg cells [5]. The immunomodulatory effects of GMSCs therefore may be used in the treatment of inflammatory and immune diseases. A variety of cytokines and chemokines, such as indoleamine 2,3-dioxygenase (IDO), nitric oxide (NO), prostaglandin E2 (PGE2), tumor necrosis factor-inducible gene 6 (TSG-6), and transforming growth factor beta (TGF-β), have been identified as regulators of MSC-based immunomodulation [1, 8–13]. However, the precise underlying mechanisms remain to be elucidated.

Metabolic alterations by H_2_S play important roles in health and the development of disease [14]. Several studies have reported that H_2_S regulates the proliferation and differentiation of mesenchymal stem cells (MSCs) [15–17]. However, the role of H_2_S in the immunomodulatory capacity of GMSCs remains to be elucidated.

In this study, we showed that H_2_S was required to maintain the immunomodulatory effect of GMSCs, which is mediated by promoting Fas/FasL coupling-induced T-cell apoptosis.

## Methods

### Mice

C57BL/6, B6.129P2-Cbstm1Unc/J, and *Cbs*^*+/****−***^ mice were purchased from Jackson Laboratory (Sacramento, CA, USA). All animal experiments were performed under institutionally approved protocols for the use of animal research at University of Pennsylvania (IACUC# 805478) and Peking University (#LA2012–65).

### Antibodies and reagents

#### Antibodies

Unconjugated MCP-1, Fas, and FasL antibodies were purchased from Santa Cruz Biotechnology (Santa Cruz, CA, USA). Anti-CD105-PE, anti-CD146-PE, anti-CD90-PE, anti-CD73-PE, anti-CD34-PE, anti-CD4-PerCP, anti-CD25-APC, anti-CD3ε, anti-CD28, and anti-CD45-PE antibody were purchased from BD Bioscience (San Jose, CA, USA). Anti-Foxp3-PE and IL-17-PE antibodies were purchased from eBioscience (San Diego, CA, USA). Anti-β-actin antibody was purchased from Sigma-Aldrich Corporation (St. Louis, MO, USA). Unconjugated anti-cystathionine β-synthase (CBS) and cystathionine γ-lyase (CSE) were purchased from Abcam Inc. (Cambridge, MA, USA).

#### Reagents

NaHS was purchased from Sigma-Aldrich. CBS, CSE, and MCP-1 siRNA were purchased from Santa Cruz Biotechnology.

### Isolation and culture of GMSCs

Gingival tissues from the mouse mandibular molar region were gently separated, minced, and digested with solution containing 2 mg/ml collagenase type I (Worthington Biochemical, Freehold, NJ, USA) and 4 mg/ml dispase II (Roche Diagnostics, Indianapolis, IN, USA) in phosphate-buffered saline (PBS) for 1 h at 37 °C. The cells were then passed through a 70-μm strainer (BD Biosciences, Franklin Lakes, NJ, USA) to obtain single cells. The single cell suspensions were cultured with α-Minimum Essential Medium (MEM) (Invitrogen, Carlsbad, CA, USA) supplemented with 20% fetal bovine serum (FBS), 2 mM l-glutamine (Invitrogen), 55 μM 2-mercaptoethanol (Invitrogen), 100 U/ml penicillin, and 100 μg/ml streptomycin (Invitrogen) and passaged, as reported previously [6]. Passage 2 of the GMSCs was used for further study.

### Isolation of mouse bone marrow mesenchymal stem cells

Bone marrow cells were flushed out from the bone cavities of femurs and tibias with 2% heat-inactivated FBS (Equitech-Bio, Kerrville, TN, USA) in PBS. Single cell suspensions of all nuclear cells were obtained by passing through a 70-μm cell strainer (BD Biosciences). All nuclear cells were seeded into 100-μm culture dishes (Corning, Corning, NY, USA) and initially incubated for 48 h at 37 °C in 5% CO_2_. To eliminate the nonadherent cells, the cultures were washed twice with PBS. The attached cells were cultured for 16 days. The BMMSCs were cultured with α-MEM supplemented with 20% FBS, 2 mM l-glutamine (Invitrogen), 55 mM 2-mercaptoethanol (Invitrogen), 100 U/ml penicillin, and 100 mg/ml streptomycin (Invitrogen).

### T-lymphocyte isolation

Mouse T cells and CD4^+^CD25^−^ T lymphocytes were isolated from mouse total spleen cells using a magnetic sorting Pan T and CD4^+^CD25^+^ regulatory T-cell isolation kit (Miltenyi Biotec, Auburn, CA, USA), according to the manufacturer’s instructions.

### T cells cocultured with GMSCs

Mouse T cells and CD4^+^CD25^−^ T cells (1 × 10^6^ cells per well) were precultured in 24-well multiplates using Dulbecco’s Modified Eagle’s Medium (Lonza, Allendale, NJ, USA) with 10% heat-inactivated FBS, 50 μM 2-mercaptoethanol, 10 mM HEPES (Sigma-Aldrich), 1 mM sodium pyruvate (Sigma-Aldrich), 1% nonessential amino acids (Lonza), 2 mM l-glutamine, 100 U/ml penicillin, and 100 mg/ml streptomycin in the presence of plate-bound anti-CD3ε antibody (2 μg/ml) and soluble anti-CD28 antibody (2 μg/ml) for 2–3 days. For Treg cell differentiation, recombinant human TGF-β1 (2 ng/ml) (R&D Systems, Minneapolis, MN, USA) and IL-2 (2 ng/ml) (R&D Systems) were added. For Th17 induction, recombinant human TGF-β1 (2 ng/ml) and IL-6 (50 ng/ml) (R&D Systems) were added. GMSCs were seeded into 24-well multiplates and incubated for 12 h. The activated T lymphocytes (0.5 × 10^6^ cells per well) were then loaded on the GMSCs and cocultured either in cell–cell contact or in a transwell coculture system. For T-cell apoptosis, after 12 h or 3 days, floating cells were harvested for flow cytometry, respectively.

For the T-cell migration assay, a transwell coculture system was used. PKH26-stained GMSCs (1 × 10^4^ cells) were seeded on the lower chamber of a 12-well culture plate (Corning) with a transwell and incubated for 12 h. The CFSE (10 μM)-labeled active T cells were loaded onto the upper chamber of the transwell and cocultured for 24 h. The culture transwells were then observed acquired using a laser-scanning microscope (LSM510; Zeiss). The green-labeled T-cell number was counted and normalized by the red-labeled number of GMSCs in five representative images as reported previously [18].

### Immunofluorescent staining

GMSCs were seeded onto a four-well chamber slide and incubated for 12 h at 37 °C in 5% CO_2_. The slides were fixed in 4% paraformaldehyde, followed by 0.01% Triton X-100 treatment for 10 min. The slides were then blocked with normal serum matched to secondary antibodies for 1 h, followed by incubation with the specific or isotype-matched antibodies (1:100) overnight at 4 °C. The slides were then treated with Rhodamin/FITC-conjugated secondary antibodies (1:200; Jackson Immuno Research, West Grove, PA, USA) for 1 h under 20–25 °C in the dark and mounted using VECTASHIELD® Mounting Medium containing 4′6-diamidino-2-phenylindole (DAPI) (Vector Laboratories, Burlingame, CA, USA). The slides were then observed and images acquired using a laser-scanning microscope (LSM510; Zeiss) and the images were processed with LSM 5 Release 4.2 software after acquisition.

### Western blot analysis

Cells were lysed in M-PER® mammalian protein extraction reagent (Pierce Chemical, Rockford, IL, USA). Total proteins (20 μg) were applied and separated on 4–12% NuPAGE® gels (Invitrogen) and transferred to Immobilon™-P membranes (Millipore, Burlington, MA, USA). The membranes were blocked with 5% nonfat dry milk and 0.1% Tween-20 for 1 h, followed by incubation with the primary antibodies (1:100–1000 dilution) at 4 °C overnight. They were then treated with horseradish peroxidase-conjugated rabbit or mouse IgG (1:10,000; Santa Cruz) for 1 h, enhanced with a SuperSignal® West Pico Chemiluminescent Substrate (Pierce Chemical), and exposed to Biomax MR film (Kodak, Rochester, NY, USA).

### Quantitative PCR

For quantitative PCR (qPCR) analysis, total RNA was extracted with an RNeasy Mini kit (Qiagen, Valencia, CA, USA), and cDNA was prepared using a SuperScript® III Reverse Transcriptase (RT) kit (Invitrogen). qPCR was performed using SYBR® Green Supermix (Bio-Rad, Hercules, CA, USA) on a Bio-Rad CFX96 Real Time System, according to the manufacturer’s instructions.

### Enzyme-linked immunosorbent assay

Culture supernatants from GMSCs were collected. The samples were centrifuged and used for MCP-1 enzyme-linked immunosorbent assay (ELISA) analysis (R&D Systems), according to the manufacturer’s instructions.

### Measurement of H_2_S

Cell culture supernatants were mixed with 0.25 ml Zn acetate (1%) and 0.45 ml water for 10 min at room temperature. Tricloroacetic acid (TCA) (10%; 0.25 ml) was then added and centrifuged (14,000 × *g*, 10 min, 4 °C). The supernatant was then collected and mixed with *N*,*N*-dimethyl-*p*-phenylenediamine sulfate (20 μM) in 1.2 M HCl and FeCl_3_ (30 μM) in 1.2 mol/l HCl. After 20 min, absorbance was measured at 650 nm.

### Flow cytometry analysis

The following antibodies were used for surface staining: anti-mouse CD146-PE, CD90-PE, CD105-PE, CD73-PE, CD34-PE, CD45-PE, CD4-Percp, CD3-APC, and CD25-APC. Staining of intracellular cytokines or transcription factors was performed using Foxp3 staining buffer (eBioscience) according to the manufacturer’s instructions. Anti-mouse Foxp3-PE and IL-17-PE were used for intracellular staining. The Annexin V-7AAD apoptosis kit (BD Biosciences) was used for analysis of T-cell apoptosis. The data were acquired using the FACS^calibur^ platform (BD Biosciences).

### Overexpression of FasL in GMSCs

For overexpression of FasL, 293 T cells for lentivirus production were plated in a 1-cm dish and cultured until 80% confluent. The plasmids were mixed in proper proportions (5:3:2) with gene expression vectors (Addgene17620), psPAX (Addgene12260), and pCMV-VSV-G (Addgene8454), and mixed in opti-MEM with Lipofectamine LTX (Invitrogen) according to the manufacturer’s instructions. The EGFP expression plasmid (Addgene17618) was used as a control. The supernatant was collected 24 and 48 h after transfection and filtered through a 0.45-μm filter to remove cell debris. For infection, the supernatant containing the lentivirus was added into the GMSC culture in the presence of 4 μg/ml polybrene and the transgenic FasL expression was validated by observation of green fluorescent protein [19].

### Knockdown assay in GMSCs

For knockdown of MCP-1 expression in GMSCs, siRNA transfection was performed according to the manufacturer’s instructions. Fluorescein-conjugated control siRNA was used as a control and as a method of evaluating transfection efficiency. All siRNA products were purchased from Santa Cruz Biotechnology (Dallas, TX, USA).

### GMSC infusion in acute colitis mice

Acute colitis was induced by administering 3% (w*/*v) dextran sulfate sodium (DSS) (molecular weight 36,000–50,000 Da; MP Biochemicals, France) through drinking water, and mice were fed ad libitum for 10 days. GMSCs or NaHS-pretreated GMSCs were infused (0.2 × 10^6^ cells) into colitis mice (*n* = 6) intravenously at day 3 after feeding DSS water. In the control group, mice were given PBS (*n* = 6). Samples from all mice were harvested at day 10 after the DSS water was provided. Induced colitis was evaluated as described previously [20].

### Statistical analysis

Comparisons between two groups were analyzed using the two-tailed Student’s *t* test using SPSS 18.0 statistical software. Comparisons between more than two groups were analyzed using one-way analysis of variance (ANOVA). A Bonferroni method was used to consider multiple comparisons. *P* < 0.05 was considered significant.

## Results

### Mouse GMSCs produced H_2_S

**Fig. 1:**
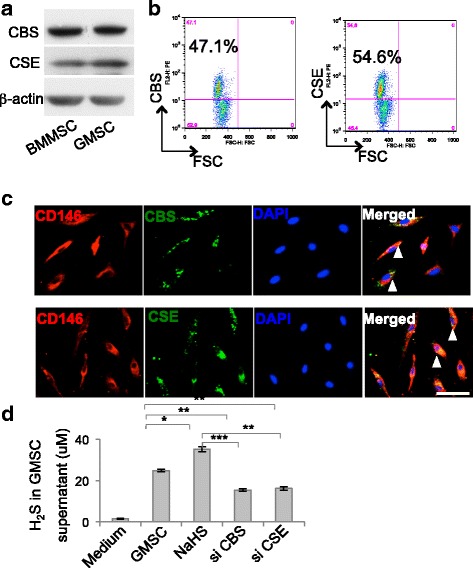
GMSCs produced H_2_S. **a** GMSCs expressed CBS and CSE, as assessed by western blot analysis. **b** Flow cytometry analysis showed 47.1% and 54.6% of GMSCs positive for CBS and CSE, respectively. **c** GMSCs positive for both CD146 and CBS or CSE, as assessed by immunofluorescence staining. **d** H_2_S donor NaHS treatment elevated H_2_S production in culture supernatant, while CBS siRNA or CSE siRNA treatment downregulated H_2_S concentrations (*n* = 5). *P < 0.05, **P < 0.01, ***P < 0.001. Scale bar: 20 μm. Error bars: mean ± SD. All experimental data verified in at least three independent experiments. CBS cystathionine β-synthase, CSE cystathionine γ-lyase, GMSC gingiva-derived mesenchymal stem cell, BMMSC bone marrow mesenchymal stem cell, FSC forward scatter, DAPI 4′6-diamidino-2-phenylindole, H_2_S hydrogen sulfide, si small interfering

We found that GMSCs isolated from mouse gingiva expressed the main catalytic enzymes of H_2_S synthesis, cystathionine β-synthase (CBS), and cystathionine γ-lyase (CSE), as did BMMSCs (Fig. 1a and Additional file 1: Figure S1A). Flow cytometry revealed that 47.1% and 54.6% of GMSCs were positive for CBS and CSE, respectively (Fig. 1b). GMSCs were positive for both CD146 and CBS or CSE (Fig. 1c). GMSCs produced H_2_S in the culture supernatant, which could be upregulated by H_2_S donor NaHS treatment and downregulated by CBS or CSE siRNA treatment (Fig. 1d and Additional file 1: Figure S1B). These data all supported the production of H_2_S by GMSCs.

### H_2_S was essential for GMSCs to induce T-cell apoptosis

**Fig. 2:**
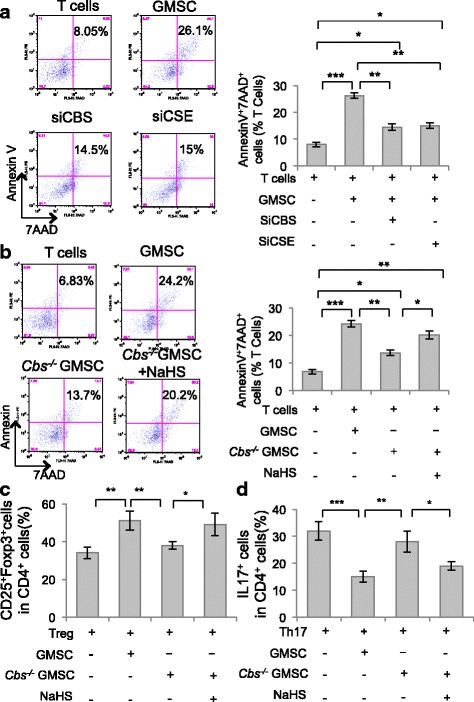
H_2_S was required for GMSCs to induce T-cell apoptosis. **a** In GMSC and T-cell coculture, GMSCs induced T-cell apoptosis, while CBS or CSE siRNA treatment attenuated GMSC-induced T-cell apoptosis, as assessed by flow cytometry (*n* = 5). **b** *Cbs*^*−/−*^ GMSCs induced less T-cell apoptosis compared with control GMSCs, and treatment with NaHS elevated T-cell apoptosis induced by *Cbs*^*−/−*^ GMSCs (*n* = 5). **c** GMSCs promoted T-cell differentiation into Treg cells induced in their polarized condition. *Cbs*^*−/−*^ GMSCs showed decreased capacity to promote Treg cell differentiation, while NaHS treatment partially restored Treg cell polarization induced by *Cbs*^*−/−*^ GMSCs (*n* = 5), as assessed by flow cytometry. **d** GMSCs inhibited T-cell differentiation into Th17 cells in a Th17 cell polarization condition. *Cbs*^*−/−*^ GMSCs showed a decreased capacity to inhibit Th17 cell differentiation, while NaHS treatment partially restored the capacity of inhibition of Th17 cell polarization by *Cbs*^*−/−*^ GMSCs, as assessed by flow cytometry (*n* = 5). *P < 0.05, **P < 0.01, ***P < 0.001. Error bars: mean ± SD. All experimental data verified in at least three independent experiments. CBS cystathionine β-synthase, CSE cystathionine γ-lyase, GMSC gingiva-derived mesenchymal stem cell, si small interfering

To analyze the immunomodulation of GMSCs, we employed GMSC and T-cell coculture systems and found that GMSCs induced T-cell apoptosis, as reported previously [1]. Both the early (AnnexinV^+^/7AAD^−^ cells) and later (AnnexinV^+^/7AAD^+^ cells) T-cell apoptosis induced by GMSCs decreased after CBS or CSE siRNA treatment (Fig. 2a and Additional file 1: Figure S2A). Moreover, GMSCs derived from *Cbs*^*−/−*^ mice induced less T-cell apoptosis than control ones. This defect of *Cbs*^*−/−*^ GMSCs could be partially restored by NaHS treatment (Fig. 2b and Additional file 1: Figure S2B). MSCs have been reported to play a role in immunomodulation by promoting Treg cell differentiation and inhibiting Th17 cell differentiation [21]. Here we revealed that GMSCs promoted Treg cell polarization and inhibited Th17 polarization in a coculture system, and that these effects were attenuated by CBS or CSE siRNA treatment (Additional file 1: Figure S2C, D). Furthermore, *Cbs*^*−/−*^ GMSCs showed a decreased capacity to promote Treg cell differentiation and inhibit Th17 cell differentiation compared with control GMSCs, and these abilities were restored by NaHS treatment (Fig. 2c, d). These results suggested that treatment of GMSCs by H_2_S induced T-cell apoptosis and regulated T-helper cell polarization.

### Immunomodulation of GMSCs via Fas/FasL-mediated T-cell apoptosis

**Fig. 3:**
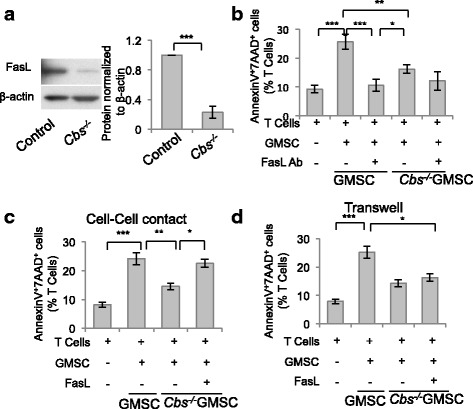
H_2_S was required to maintain FasL expression in GMSCs. **a** FasL expression decreased in *Cbs*^*−/−*^ GMSCs compared with control GMSCs as analyzed by western blot analysis. **b** *Cbs*^*−/−*^ GMSCs induced less T-cell apoptosis compared with control cells. Fas-neutralized antibody treatment attenuated the ability of GMSCs to induce T-cell apoptosis (*n* = 5). **c**, **d** FasL overexpression restored the decreased capacity of *Cbs*^*−/−*^ GMSC-inducing T cells in a cell–cell contact coculture system, but not in a transwell coculture system (*n* = 5). *P < 0.05, **P < 0.01, ***P < 0.001. Error bars: mean ± SD. All experimental data verified in at least three independent experiments. GMSC gingiva-derived mesenchymal stem cell, FasL Fas ligand, Ab antibody

**Fig. 4:**
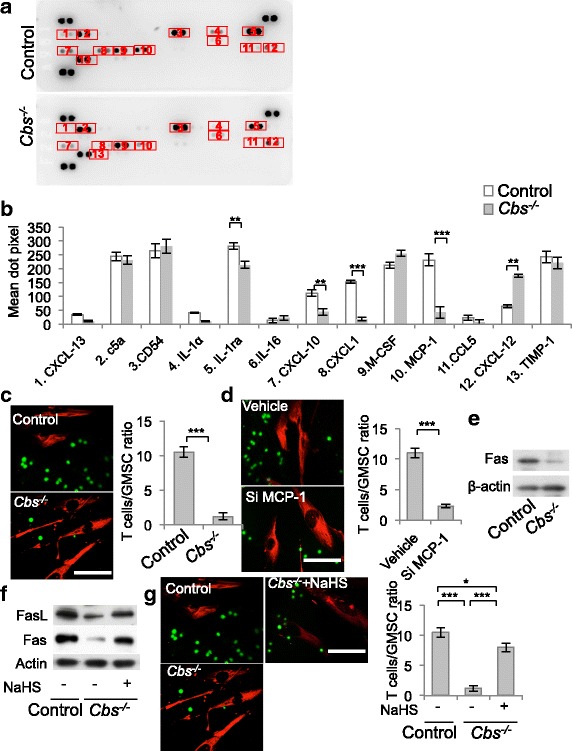
GMSCs achieved immunomodulation through Fas/FasL-mediated T-cell apoptosis. **a**, **b** Cytokine profiles of control and *Cbs*^*−/−*^ GMSCs, assessed by cytokine array. **c** GMSCs recruited T cells in transwell coculture, and *Cbs*^*−/−*^ GMSCs recruited less T cells compared with controls (*n* = 5). **d** Capacity of GMSCs recruiting T cells was attenuated after MCP-1 siRNA treatment (*n* = 5). **e**, **f** Fas expression decreased in *Cbs*^*−/−*^ GMSCs compared with control GMSCs, while decreased Fas expression in *Cbs*^*−/−*^ GMSCs was partially restored by NaHS treatment. **g** NaHS treatment restored the attenuated capacity of recruiting T cells by *Cbs*^*−/−*^ GMSCs, as assessed by immunofluorescence staining (*n* = 5). *P < 0.05, **P < 0.01, ***P < 0.001. Scale bar: 10 μm. All experimental data verified in at least three independent experiments. CXCL chemokine (C–X–C motif) ligand, IL interleukin, M-CSF macrophage colony stimulating factor, MCP-1 monocyte chemotactic protein 1, GMSC gingiva-derived mesenchymal stem cell, si small interfering, FasL Fas ligand

To explore the underlying mechanism of how H_2_S regulates the immunomodulatory effects of GMSCs, we analyzed the expression of FasL, a key member of the apoptosis pathway, in GMSCs and found that FasL expression was decreased in *Cbs*^*−/−*^ GMSCs compared with controls (Fig. 3a). When cocultured with T cells, GMSCs induced T-cell apoptosis, which was blocked by treatment with FasL neutralizing antibody (Fig. 3b). Furthermore, when we cocultured T cells and GMSCs in cell–cell contact, the compromised capacity of *Cbs*^*−/−*^ GMSCs to induce T-cell apoptosis was restored by overexpression of FasL (Fig. 3c and Additional file 1: Figure S3). The results suggested that FasL was essential in GMSC-induced T-cell apoptosis. However, when GMSCs were cocultured with T cells in a transwell system, FasL overexpression failed to enhance the decreased immunomodulation of *Cbs*^*−/−*^ GMSCs (Fig. 3d), which indicated that soluble factors may be involved in H_2_S-mediated immunomodulation by GMSCs. We then compared the cytokine profiles between control and *Cbs*^*−/−*^ GMSCs using a cytokine array and found that the chemokines CXCL-10, CXCL-1, and MCP-1 were significantly decreased in *Cbs*^*−/−*^ GMSCs (Fig. 4a, b). We next found that fewer activated T cells migrated to *Cbs*^*−/−*^ GMSCs compared with control GMSCs in a transwell coculture system (Fig. 4c). Monocyte chemotactic protein 1 (MCP-1), a T-cell chemoattractant cytokine, has been reported to play a crucial role in MSC-mediated T-cell apoptosis [18, 22]. We knocked down MCP-1 using siRNA and found that fewer activated T cells migrated to GMSCs after this treatment (Fig. 4d). The death receptor, Fas, regulates the secretion of cytokines and exosomes including MCP-1 and C–X–C motif chemokine 10 (CXCL-10) [18]. We found that Fas expression was decreased in *Cbs*^*−/−*^ GMSCs (Fig. 4e and Additional file 1: Figure S4A). Fas and FasL expression in *Cbs*^*−/−*^ GMSCs were upregulated by treatment with NaHS (Fig. 4e). MCP-1 expression was elevated in *Cbs*^*−/−*^ GMSCs compared with controls, and this elevated expression was attenuated by NaHS treatment (Additional file 1: Figure S4B–D). MCP-1 secretion of *Cbs*^*−/−*^ GMSCs in the culture supernatant was reduced relative to control GMSCs, and could be upregulated by NaHS treatment (Additional file 1: Figure S4E). Moreover, NaHS treatment upregulated the expression of Fas and FasL in *Cbs*^*−/−*^ GMSCs (Fig. 4f and Additional file 1: Figure S4F) and increased the number of activated T cells that migrated to *Cbs*^*−/−*^ GMSCs (Fig. 4g). Taken together, these results indicated that T-cell recruitment by Fas-mediated MCP-1 played an important role in GMSC-induced T-cell apoptosis.

### H_2_S promoted the therapeutic effects of GMSCs in the treatment of colitis

**Fig. 5:**
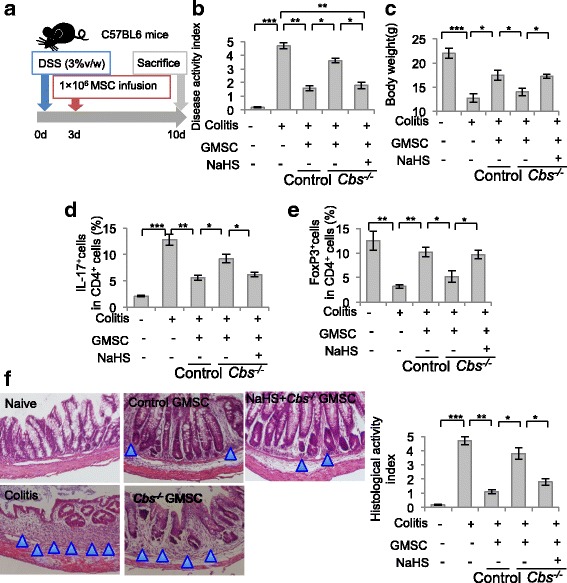
H_2_S was required to maintain GMSC immunomodulation in vivo. **a** Schematic of GMSC infusion in DSS-induced experimental colitis mice. **b** GMSC infusion reduced the disease activity index (DAI) induced by DSS administration, while *Cbs*^*−/−*^ GMSCs failed to reduce the DAI, and NaHS pretreatment partially restored the reduction of DAI by *Cbs*^*−/−*^ GMSCs in colitis mice. **c**–**f** GMSC infusion restored mice body weight loss, Th17 cell elevation, Treg cell decrease, and histological activity increase induced by DSS, while *Cbs*^*−/−*^ GMSCs failed to restore these clinical symptoms and histological observations in mice. NaHS pretreatment partially increased the capacity of *Cbs*^*−/−*^ GMSCs to restore mice body weight loss, Th17 cell elevation, Treg cell decease, and histological activity increase induced by DSS. Blue triangle, inflammatory cells. *P < 0.05, **P < 0.01, ***P < 0.001. Scale bar: 100 μm (*n* = 6). All experimental data verified in at least three independent experiments. d days, DSS dextran sulfate sodium, MSC mesenchymal stem cell, GMSC gingiva-derived mesenchymal stem cell, IL interleukin

**Fig. 6:**
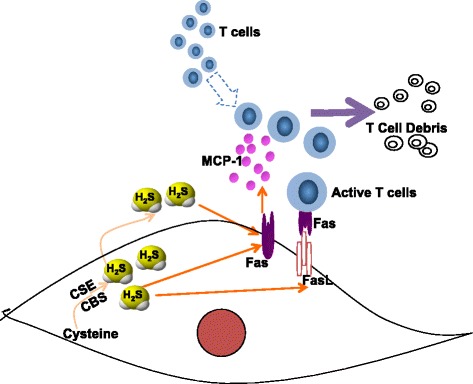
Schematic showing that H_2_S metabolism regulates the immunomodulatory capacity of GMSCs via Fas/FasL coupling-induced T-cell apoptosis. MCP-1 monocyte chemotactic protein 1, FasL Fas ligand, H_2_S hydrogen sulfide, CBS cystathionine β-synthase, CSE cystathionine γ-lyase

To further study the role of H_2_S in immunomodulation by GMSCs, we compared the immunotherapeutic effects between control and *Cbs*^*−/−*^ GMSCs in mice with dextran sulfate sodium (DSS)-induced colitis. Control GMSCs, *Cbs*^*−/−*^ GMSCs, or NaHS-treated GMSCs (0.2 × 10^6^ cells) were systemically infused into mice with colitis at day 3 after 3% DSS treatment (Fig. 5a). The disease activity index (DAI), determined by the presence of sustained weight loss and bloody diarrhea/loose feces, was elevated in the mice with colitis compared with the control group. After infusion of control GMSC or NaHS-pretreated *Cbs*^*−/−*^ GMSCs, but not *Cbs*^*−/−*^ GMSCs, the DAI score decreased (Fig. 5b). Infusion of control GMSCs, but not *Cbs*^*−/−*^ GMSCs, partially restored the reduced body weight of mice with DSS-induced colitis. NaHS pretreatment enhanced the ability of *Cbs*^*−/−*^ GMSCs to restore the reduced body weight of mice with colitis (Fig. 5c). Decreased numbers of Treg cells and elevated numbers of Th17 cells were observed in the mice with colitis at day 10 post DSS administration (Fig. 5d, e). Control GMSC infusion, but not *Cbs*^*−/−*^ GMSC infusion, increased the number of Treg cells and decreased that of Th17 cells. NaHS pretreatment increased the capacity of *Cbs*^*−/−*^ GMSC infusion to increase the number of Treg cells and decrease that of Th17 cells (Fig. 5d, e). Infiltration of inflammatory cells, impairment of colon structures, and the absence of an epithelial layer were observed in histological analysis of mice with colitis. Infusion of control GMSCs or NaHS-pretreated *Cbs*^*−/−*^ GMSC, but not *Cbs*^*−/−*^ GMSC, partially restored the histological appearance of the colon in DSS-induced colitis mice, eliminating the inflammatory cells and showing evidence for recovery of the epithelial structure (Fig. 5f). Together, these results confirmed that H_2_S is important for maintaining the immunomodulatory effects of GMSCs (Fig. 6).

## Discussion

H_2_S has been reported to play an important role in MSCs by enhancing the proliferation and differentiation [16]. Here we showed, for the first time, that a physiological level of H_2_S was required to maintain the immunomodulatory capacity of GMSCs. H_2_S deficiency in GMSCs led to reduced T-cell apoptosis when cocultured with GMSCs. Furthermore, H_2_S-deficient GMSCs showed compromised therapeutic effects on experimental colitis in mice.

Fas/FasL interaction induces oligomerization and aggregation of the Fas receptor, leading to apoptosis after protein–protein interactions. A previous study indicated that MSC-induced immune tolerance was associated with FasL-triggered T-cell apoptosis via the Fas/FasL death pathway [18] In the present study, we showed that H_2_S was required to maintain the immunoregulatory function of GMSCs by enhancing the expression of FasL. Conversely, H_2_S-deficient GMSCs with decreased expression of FasL showed a significantly decreased immunomodulation. Cell–cell contact was essential for Fas/FasL-mediated T-cell apoptosis. In our coculture system, FasL overexpression rescued the decrease in T-cell apoptosis induced by H_2_S-deficient GMSCs in a cell–cell contact manner, but not in a transwell chamber. These results indicated that there may be some soluble factors in addition to FasL participating in the regulation of immunomodulation of GMSCs. Furthermore, we showed that the expression of chemokines associated with cell trafficking, including CXCL-10, CXCL-1, and MCP-1, were significantly decreased in H_2_S-deficient GMSCs compared with control GMSCs. The MCP-1/CCR2 signaling axis has been implicated in the trafficking of T cells, macrophages, and myeloid-derived suppressor cells in tumors and immune disorders [23–25]. It has been reported that MCP-1 produced by MSCs mobilized macrophages to modulate the Th1/Th17 immune response, which contributed to the therapeutic effects of MSCs on experimental autoimmune encephalitis (EAE) or experimental autoimmune uveitis (EAU) [24, 26, 27]. MCP-1 secreted by MSCs was also reported to recruit activated T cells to MSCs to cause immune tolerance [18]. Here we showed that MCP-1 produced by GMSCs recruited activated T cells to GMSCs, which in turn resulted in GMSC-induced T-cell apoptosis through the Fas/FasL death pathway.

Recent studies have shown that Fas controls exosome release by MSCs via epigenetic regulation [28]. Here we found that Fas expression, which was regulated by the levels of H_2_S in GMSCs, controlled MCP-1 secretion. We also found that Fas-mediated MCP-1 secretion in GMSCs stimulated the migration of activated T cells in a transwell chamber, which reflected what happens in vivo when T cells migrate to transplanted GMSCs. FasL expressed in GMSCs then induced T-cell apoptosis via the Fas/FasL death pathway in a cell–cell dependent manner. Furthermore, the level of H_2_S played a critical role in promoting both Fas and FasL expression to cause immune tolerance. However, the underlying mechanism of Fas-dependent control of MCP-1 secretion needs to be studied further.

The large number of antigens present in commensal bacteria of the oral microflora makes the induction of immune tolerance in oral mucosa unique. Disturbances of the immune network contribute to the development of gingivitis and periodontitis [14]. H_2_S plays crucial roles in regulating the immunomodulation of GMSCs and H_2_S donor treatment could elevate the immunomodulation of GMSCs. Manipulation of H_2_S levels may therefore be an alternative choice for preventing or treating inflammation and infections, including colitis and periodontal disease.

## Conclusions

The level of H_2_S controlled the immunomodulatory effect of GMSCs, which was mediated by Fas/FasL coupling-induced T-cell apoptosis (Fig. 6).

## Additional file

Additional file 1: is **Figure S1** showing mouse GMSCs produced H_2_S, **Figure S2** showing H_2_S is required in GMSCs to induce T-cell apoptosis, **Figure S3** showing efficacy of FasL overexpression, as assessed by western blot analysis, and **Figure S4** showing H_2_S promoted T cells migrating to GMSCs via promoting MCP-1 secretion. (PDF 1802 kb)

## Abbreviations

CBS: Cystathionine β-synthase
CSE: Cystathionine γ-lyase
CXCL-1: Chemokine (C–X–C motif) ligand 1
CXCL-10: Chemokine (C–X–C motif) ligand 10
DSS: Dextran sulfate sodium
EAE: Autoimmune encephalitis
EAU: Experimental autoimmune uveitis
GMSC: Mesenchymal stem cell derived from gingiva
H_2_S: Hydrogen sulfide
IDO: Indoleamine 2,3-dioxygenase
MCP-1: Monocyte chemotactic protein 1
MSC: Mesenchymal stem cell
NO: Nitric oxide
PBMC: Peripheral blood mononuclear cell
PGE2: Prostaglandin E2
TGF-β: Transforming growth factor beta
TSG-6: Tumor necrosis factor-inducible gene 6
