# Supplementary material for: Hydrogen sulfide promotes immunomodulation of gingiva-derived mesenchymal stem cells via the Fas/FasL coupling pathway
Source: Stem Cell Res Ther. 2018 Mar 9;9:62. doi: 10.1186/s13287-018-0804-6 (PMC5845196; doi:10.1186/s13287-018-0804-6)
Supplement: Supplementary file 1 — is Figure S1 showing mouse GMSCs produced H2S, Figure S2 showing H2S is required in GMSCs to induce T-cell apoptosis, Figure S3 showing efficacy of FasL overexpression, as assessed by western blot analysis, and Figure S4 showing H2S promoted T cells migrating to GMSCs via promoting MCP-1 secretion. (PDF 1802 kb) [file 13287_2018_804_MOESM1_ESM.pdf]

ADDITIONAL FILE LEGEND

Figure S1 Mouse GMSCs produced H_2S. (A) Stem cell markers profile of GMSCs analyzed by flow cytometry. (B) The efficacy of CBS and CSE siRNA analyzed by western blot. All experimental data were verified in at least three independent experiments.

Figure S2 H_2S is required in GMSCs to induced T cell apoptosis.

(A) In GMSC and T cell co-culture for 12h, GMSCs induced Annexin V^+ $7AAD^-$ early and Annexin V^+ $7AAD^+$ T cell apoptosis, while CBS or CSE siRNA treatment attenuated GMSC-induced T cell apoptosis, as assessed by flow cytometry (n=5). (B) *Cbs*$^{-/-}$ GMSCs induced less Annexin V^+ $7AAD^-$ and Annexin V^+ $7AAD^+$ T cell apoptosis compared with control GMSCs, and treatment with NaHS elevated Annexin V^+ $7AAD^-$ and Annexin V^+ $7AAD^+$ T cell apoptosis induced by *Cbs*$^{-/-}$ GMSCs (n=5), when T cells were co-cultured with GMSCs for 12h. (C) GMSCs promoted Treg cells differentiation, which were decreased after CBS or CSE siRNA treatment, as assessed by flow cytometry (. (D) GMSCs inhibited Th17 cells differentiation, which were attenuated by CBS or CSE siRNA treatment, as assessed by flow cytomotry. * $P<0.05$, ** $P<0.01$, *** $P<0.001$. Error bars: mean ± SD. All experimental data were verified in at least three independent experiments.

Figure S3 The efficacy of FasL overexpression, as assessed by western blot. All experimental data were verified in at least three independent experiments.

Supplementary Figure 4 H_2S promoted T cells migrating to GMSCs *via* promoting its MCP-1 secretion. (A) The quantification of protein expression level according to Fig.4E. (B) The efficacy of MCP-1 siRNA analyzed by qPCR. (C) MCP-1 secretion in culture supernatant was decreased after MCP-1 siRNA treatment. (D) MCP-1 expression was upregulated in H_2S deficiency compared to control ones, and NaHS treatment decreased elevated MCP-1 in *Cbs*$^{-/-}$ GMSCs, as assessed by western blot. (E) NaHS treatment restored decreased MCP-1 secretion in *Cbs*$^{-/-}$ GMSCs compared with control ones. (F) The quantification of protein expression level according to Fig.4F. * $P<0.05$, ** $P<0.01$, *** $P<0.001$. Error bars: mean ± SD. All experimental data were verified in at least three independent experiments.

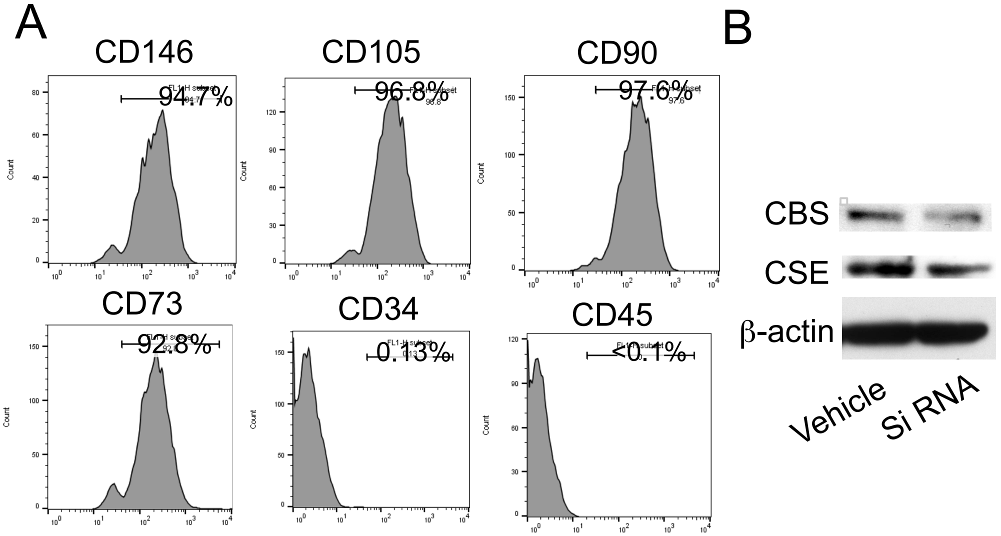
A
CD146
94.7%
CD105
96.8%
CD90
97.6%
CD73
92.8%
CD34
0.13%
CD45
<0.1%
B
CBS
CSE
β-actin
Vehicle
Si RNA

Figure S1

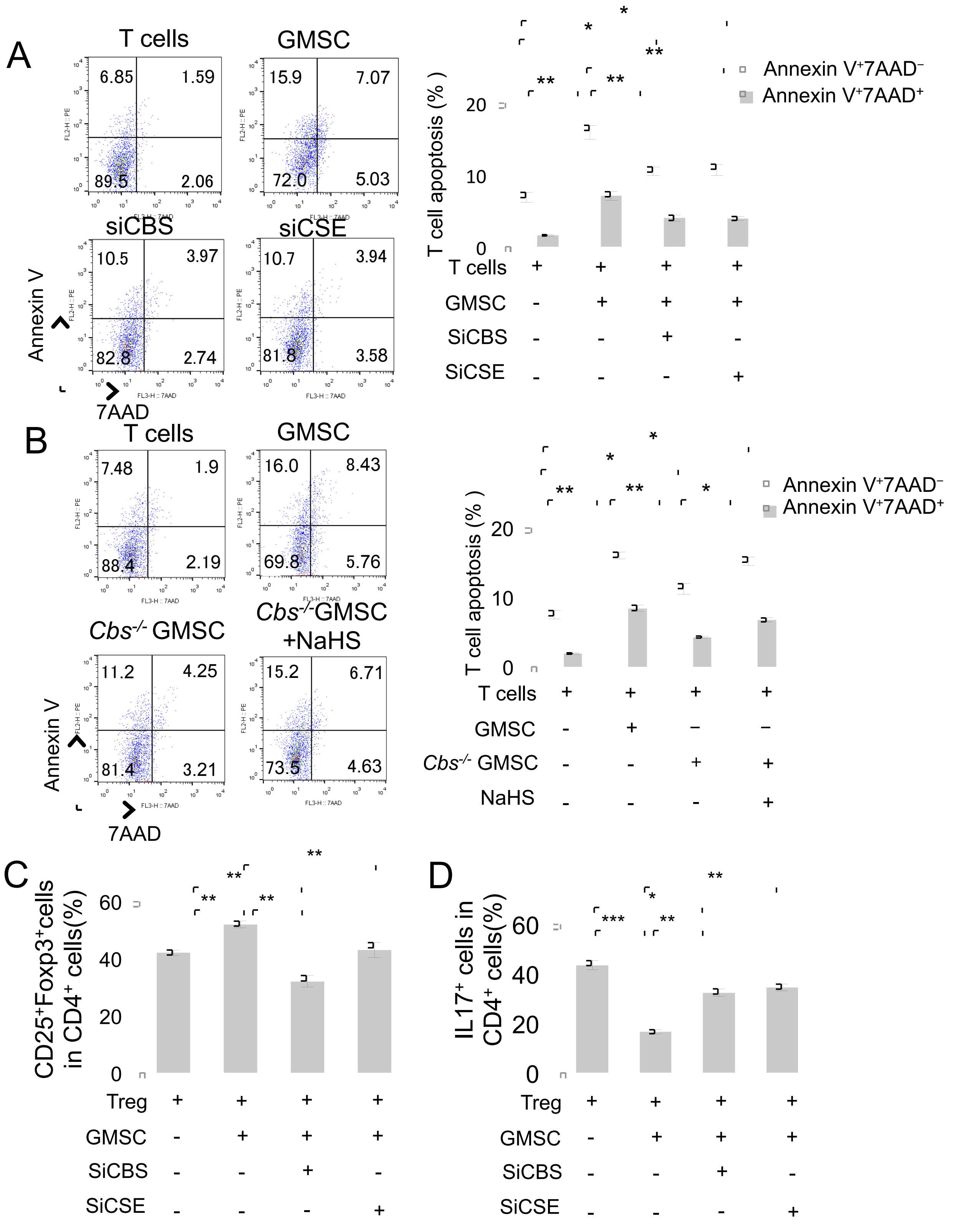
A
T cells
GMSC
6.85
1.59
89.5
2.06
15.9
7.07
72.0
5.03
siCBS
siCSE
10.5
3.97
82.8
2.74
10.7
3.94
81.8
3.58
Annexin V
7AAD
T cell apoptosis (%)
Annexin V+7AAD−
Annexin V+7AAD+
T cells
GMSC
SiCBS
SiCSE
B
T cells
GMSC
7.48
1.9
88.4
2.19
16.0
8.43
69.8
5.76
Cbs-/- GMSC
Cbs-/- GMSC +NaHS
11.2
4.25
81.4
3.21
15.2
6.71
73.5
4.63
Annexin V
7AAD
T cell apoptosis (%)
Annexin V+7AAD−
Annexin V+7AAD+
T cells
GMSC
Cbs-/- GMSC
NaHS
C
CD25+Foxp3+cells in CD4+ cells(%)
Treg
GMSC
SiCBS
SiCSE
D
IL17+ cells in CD4+ cells(%)
Treg
GMSC
SiCBS
SiCSE

Figure S2

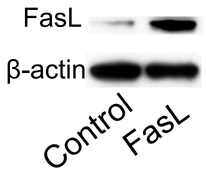

Figure S3

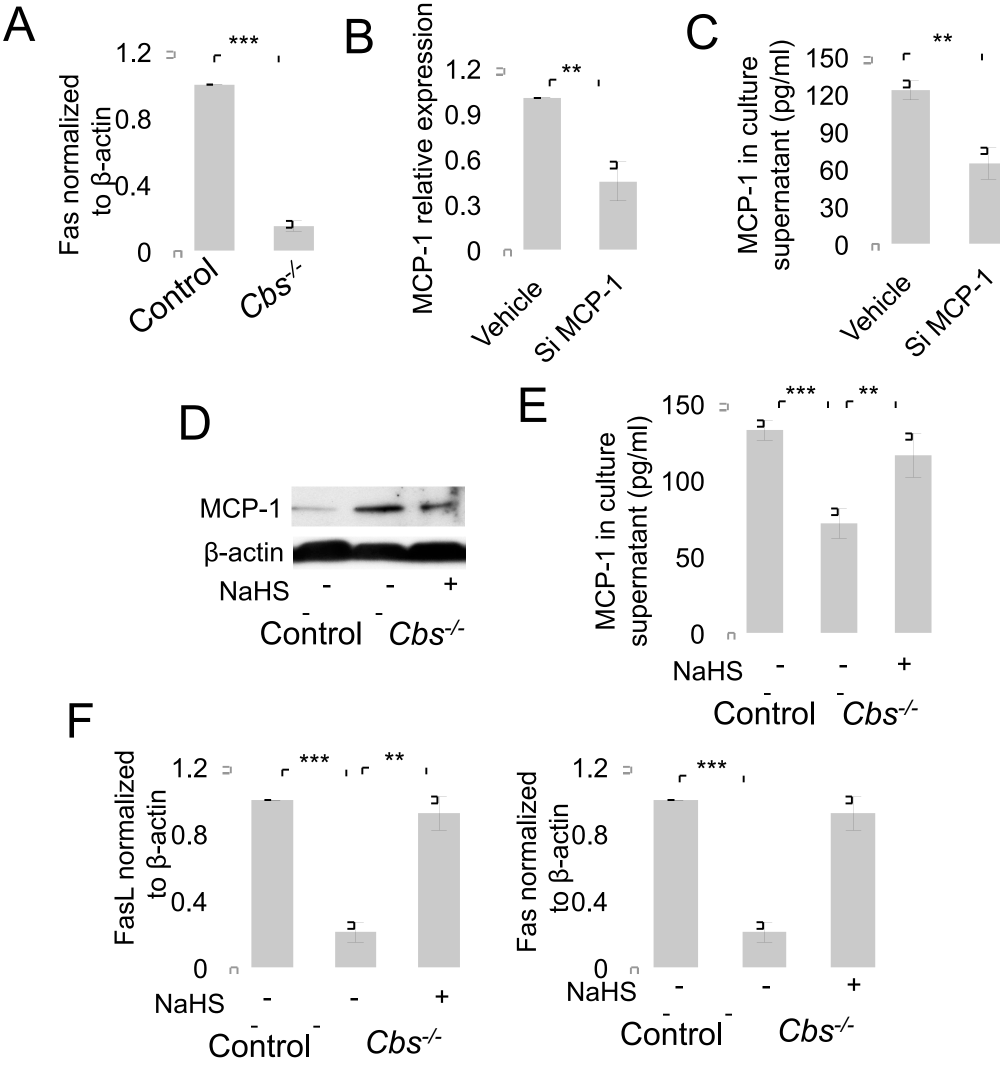
A
Fas normalized to β-actin
1.2
0.8
0.4
0
***
Control
Cbs-/-
B
MCP-1 relative expression
1.2
0.9
0.6
0.3
0
**
Vehicle
Si MCP-1
C
MCP-1 in culture supernatant (pg/ml)
150
120
90
60
30
0
**
Vehicle
Si MCP-1
D
MCP-1
β-actin
NaHS - - +
Control
Cbs-/-
E
MCP-1 in culture supernatant (pg/ml)
150
100
50
0
***
**
NaHS - - +
Control
Cbs-/-
F
FasL normalized to β-actin
1.2
0.8
0.4
0
***
**
NaHS - - +
Control
Cbs-/-
Fas normalized to β-actin
1.2
0.8
0.4
0
***
NaHS - - +
Control
Cbs-/-

Figure S4
